# Favorable clinical outcome and unique characteristics in association with *Twist1* overexpression in *de novo* acute myeloid leukemia

**DOI:** 10.1038/bcj.2015.67

**Published:** 2015-08-14

**Authors:** C-C Chen, J-Y You, J-P Gau, C-E Huang, Y-Y Chen, Y-H Tsai, H-J Chou, J Lung, M-H Yang

**Affiliations:** 1 Division of Hematology and Oncology, Department of Medicine, Chang Gung Memorial Hospital, Chiayi, Taiwan; 2 College of Medicine, Chang Gung University, Tao-Yuan, Taiwan; 3 School of Medicine, National Yang-Ming University, Taipei, Taiwan; 4 Division of Hematology and Oncology, Department of Medicine, Lotung Poh-Ai Hospital, Yilan, Taiwan; 5 Division of Hematology and Oncology, Department of Medicine, Taipei Veterans General Hospital, Taipei, Taiwan; 6 Division of Pulmonary and Critical Care Medicine, Department of Medicine, Chang Gung Memorial Hospital, Chiayi, Taiwan; 7 Institute of Clinical Medicine, National Yang-Ming University, Taipei, Taiwan; 8 Immunology Research Center, National Yang-Ming University, Taipei, Taiwan; 9 Genome Research Center, National Yang-Ming University, Taipei, Taiwan

## Abstract

Epithelial–mesenchymal transition (EMT) is a critical process for inducing stem-like properties of epithelial cancer cells. However, the role of EMT inducers in hematological malignancies is unknown. *Twist1*, an EMT inducer necessary for cell migration, has recently been found to have transcriptionally regulatory activity on the expression of *Bmi1*, and these two are capable of promoting tumorigenesis in a synergized manner. Knowing that *Bmi1* expression is essential for maintenance of leukemic stem cells, we speculate that *Twist1* might govern the pathogenesis of acute myeloid leukemia (AML) development as well. We found that upregulated *Twist1* increased *Bmi1* expression in AML and endued leukemic cells a higher proliferative potential and increased resistance to apoptosis. In primary AML samples, there was strong positive correlation between the expression levels of *Twist1* and *Bmi1*. AML patients whose leukemic blasts harbored overexpressed *Twist1* had a more aggressive clinical phenotype, but they were more likely to have a better clinical outcome after standard therapy. *In vitro* studies confirmed that *Twist1*-overexpressing leukemic cells were more susceptible to cytarabine, but not daunorubicin, cytotoxicity. Our findings suggest that, in a subset of AML patients, *Twist1* has a prominent role in the pathogenesis of the disease that leads to unique clinical phenotypes.

## Introduction

Acute myeloid leukemia (AML) is a clonal hematological disease characterized by multiple genetic anomalies resulting in altered self renewal, impaired cell differentiation, excessive proliferation and inadequate apoptosis.^1^ Although a significant portion of patients would achieve complete remission (CR) after induction chemotherapy, the outcomes for AML patients remain dismal as the majority of those attaining CR ultimately relapse. Recent advances in molecular biology have led to identification of several genetic markers with important prognostic implication in AML, such as mutations in *FLT3* (fms-like tyrosine kinase 3),^2^ *NPM1* (nucleophosmin 1)^3^ and *CEBPA* (CCAAT/enhancer binding protein alpha).^4^ These markers are adequately incorporated in clinical guidelines to predict outcome and direct therapy in AML.^5, 6^ However, a large number of AML patients do not possess these predictive markers and there remains significant discrepancy in the clinical outcome of patients within the same categorized risk group.^7^ Therefore, there is a pressing urgency that additional biomarkers with novel pathognomic indication and accurate prognostic value are needed for further refinement of AML risk categorization.

*BMI1* is a member of the Polycomb group genes that have essential roles in maintaining chromatin silencing.^8, 9^ It is critically involved in the self-renewal of hematopoietic, intestinal and neural stem cells through repression of *INK4A*–*ARF* locus.^10, 11, 12, 13^ Originally identified as a collaborating oncogene in the induction of lymphoma,^14, 15^ *Bmi1* was subsequently reported overexpressed in various human cancers.^16^ In addition to functions in normal hematopoiesis, *Bmi1* has been suggested to have a role in leukemogenesis as well.^16, 17^ In AML CD34^+^ cells, downmodulation of *Bmi1* results in impairment of long-term expansion and loss of self-renewal capacity.^18^ Studies have shown that *Bmi1* expression is elevated in leukemic blasts of AML.^19, 20^ High *Bmi1* expression is correlated with survival outcome in AML patients.^21^ These data highlight the functional importance of *Bmi1* overexpression in AML.

Epithelial–mesenchymal transition (EMT) is a process which involves in the reprogramming of epithelial cells into a mesenchymal-like cells, and the importance of EMT has been highlighted in different human cancers.^22^ Recently, EMT has been shown to generate cells with stem-like properties,^23, 24^ which is very important in the late-stage progression and metastatic colonization of epithelial cancers. However, the role of EMT inducers in hematological malignancies is largely unknown. Twist1, a bHLH (basic helix–loop–helix) transcription factor, is a well-known EMT inducer that is necessary for cell migration, tissue reorganization and morphogenesis during embryonic development.^25^ These cell functions are also required for tumor invasion and metastasis.^26^ Indeed, high levels of *Twist1* expression have been correlated with invasive/metastatic features of carcinoma of breast, stomach, prostate and nasopharyngeal cancer.^27, 28, 29, 30^ We recently demonstrated that *Twist1* could activate *Bmi1* expression via direct binding to its regulatory region.^31^ Moreover, *Twist1* and *Bmi1* are mutually essential for promoting tumor-initiating ability, and co-occupancy of *Twist1* and *Bmi1* on the regulatory regions are required to repress *p16INK4A.*^31^ These data provide a critical mechanism in which *Twist1* induces chromatin remodeling by activating *Bmi1* expression. Sporadic reports indicate the aberrant expression and deregulation of *Twist1* in myeloid neoplasms, including myelodysplastic syndrome and chronic myeloid leukemia.^32, 33^ Knowing that *Bmi1* expression is essential for maintenance and self-renewal of leukemic stem and progenitor cells, we speculate that *Twist1* might govern the pathogenesis of development of AML as well. In the current work, we aim to investigate potential interaction between Twist1 and Bmi1 in AML and dissect the prognostic relevance of *Twist1* in these patients.

## Materials and methods

### Study population and sample collection

Diagnostic bone marrow samples were collected and cryopreserved from patients diagnosed with AML. All patients gave written informed consent in accordance with the Declaration of Helsinki to participate in the study. This study was approved by the Institutional Review Board of Chang-Gung Memorial Hospital.

Overall, 41 samples were collected. All patients were treated according to institutional guidelines. Cytogenetic and molecular risk groups were categorized according to European LeukemiaNet recommendations.^5^ CR, relapse, disease-free survival and overall survival were defined according to previously proposed criteria.^34^

### Cell culture and reagents

Human AML cell lines, KG1a and THP1, were purchased from Bioresearch Collection and Research Center, Hsinchu, Taiwan. Both cells were maintained according to the distributor's recommendation. Cytarabine and daunorubicin were obtained from Pfizer (Taipei, Taiwan).

### Plasmids and transfection

Production of the pFlag-Twist plasmid has been previously described.^35^ The plasmids for small interfering RNA (siRNA) experiments, generated by inserting an oligonucleotide containing a specific siRNA target sequence directed against *Twist1* or a scrambled sequence into the pLKO_TRC005 and pLKO.1 vectors, respectively, were purchased from National RNAi Core Facility (Taipei, Taiwan). Nucleofection was performed using the Amaxa Cell Line Nucleofector Kits (Lonza Cologne AG, Cologne, Germany) according to the manufacturer's instructions. Cells were harvested after 48 h of transfection and subjected to western blotting, cytotoxic assay, apoptotic assay and immunophenotyping. The numbers of surviving cell were counted using trypan blue and recorded daily until 1 week following transfection.

### Western blotting

Western blotting analysis was performed as described previously.^36^ Antibodies against Bmi1 (Millipore, Billerica, MA, USA), Twist1 (Santa Cruz Biotechnology, Santa Cruz, CA, USA), p14 (Cell Signaling Technology, Danvers, MA, USA), p16 (Cell Signaling Technology) and Histone H3 (Santa Cruz Biotechnology) were all used in a 1/1000 dilution, and antibody against actin was used in a 1/10 000 dilution. Anti-mouse and anti-rabbit secondary antibody (both from Millipore) was used in a 1/5000 dilution.

### Growth-inhibition assay

Exponentially growing cells were cultured overnight. Growth-inhibition assay as performed previously, and the drug concentration that inhibited cell growth by 50% (IC_50_) was determined.^37^

### Immunophenotyping and apoptotic assay

Detection of cell surface antigen expression was performed as described previously.^36^ CD34, CD33, CD13 and CD14 monoclonal antibodies were purchased from Becton Dickinson, San Jose, CA, USA. All fluorescence-activated cell sorter analyses were performed on a FACSCalibur flow cytometer (Becton Dickinson). For apoptotic assay, propidium iodide and Annexin V was obtained from BD Pharmingen, San Diego, CA, USA.

### Real-time quantitative reverse transcriptase-PCR (qRT-PCR) analysis

Total RNA was extracted using TRIzol reagent according to the manufacturer's instructions (Invitrogen, Carlsbad, CA, USA) and treated with DNaseI before cDNA synthesis to remove DNA contamination. First-strand cDNA was generated using Super-Script III first-strand synthesis system as recommended by the manufacturer (Invitrogen). The expression levels of *Twist-1*, *BMI-1* and *β-actin* were measured by qRT-PCR using dual-labeled TaqMan probes (Applied Biosystem, Carlsbad, CA, USA) containing 5′ FAM (5-carboxyfluorescein) reporter dye and 3′ NFQ (nonfluorescent quencher). Primers and Taqman probes were designed by Probe Finder (Roche, Indianapolis, IN, USA; http://www.universalprobelibrary.com). The sequences are listed in Table 1. qRT-PCR was performed using the Qiagen Rotor Gene Q system (Qiagen, Hilden, Germany). For amplification of *Twist1*, the condition was 95 °C for 10 min and 50 cycles of 95 °C for 15 s and 60 °C for 1 min. For *Bmi1* amplification, the condition was 95 °C for 10 min and 50 cycles of 95 °C for 15 s, 64 °C for 30 s and 72 °C for 30 s. The β-actin-normalized data are presented as the fold change in gene expression in the treatment group compared with controls. Changes in gene expression level relative to the normal bone marrow samples were calculated using the 2^−ΔΔ^CT method.

### Statistical analysis

All assays were carried out in triplicate. Data are expressed as mean and s.d. Data analysis and compassion was performed using appropriate statistical methods. A two-sided *P*-value of <0.05 was considered statistically significant for all tests.

## Results

### Bmi1 transcripts are upregulated in AML Cells with Twist1 overexpression

**Figure 1:**
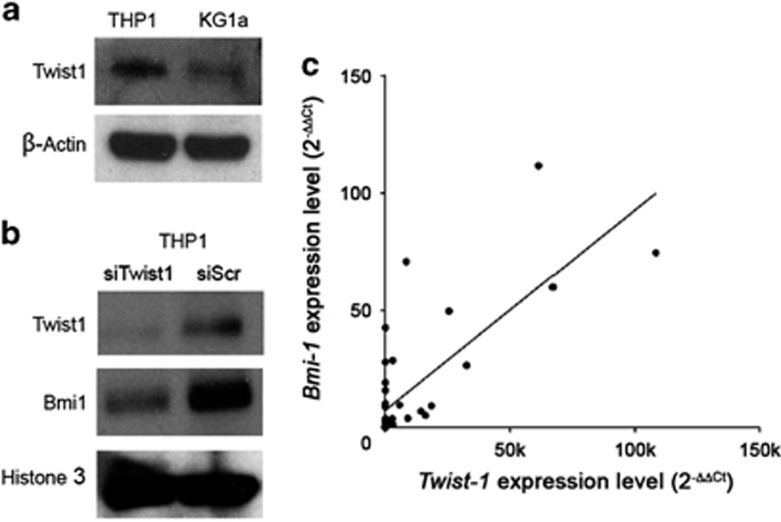
Expression of Twist1 and its correlation with Bmi1 level in AML cell lines and primary AML samples. (**a**) Western blotting results of the expression levels of Twist1 protein in the whole cell lysates of two AML cell lines, THP1 and KG1a. β-Actin was used as an internal control. Representative data from three independent experiments are presented. (**b**) Effects of *Twist1*-targeted siRNA on the protein levels of Twist1 and Bmi1 in THP1 cells. *Twist1*-overexpressing THP1 cells were transiently transfected with either *Twist1*-targeted siRNA or scrambled control for 48 h, and the nuclear lysates were examined by western blotting. Histone 3 was used as a loading control. Representative data from three independent experiments are shown. (**c**) Positive correlation between *Twist-1* and *Bmi-1* gene expression in primary AML samples examined by real-time RT-PCR (*r*=0.5662, *P*<0.0001, Pearson's correlation).

We screened several AML cell lines and found that KG1a and THP1 cells had low and high expression levels of *Twist1*, respectively (Figure 1a). To investigate whether the expressional status of *Twist1* affects the activation of *Bmi1*, we transfected THP1 cells with either *Twist1*-targeted siRNA or scrambled control. Downregulation of *Twist1* resulted in decreased expression of Bmi1 in THP1 cells (Figure 1b). To see whether these findings were clinically relevant, we checked the expression levels of *Twist1* and *Bmi1* in primary AML samples by qRT-PCR. As shown in Figure 1c, there was a good positive correlation between the expression levels of *Twist1* and *Bmi1* in the leukemic blasts of our AML patients (correlation coefficient *r*=0.5662, *P*<0.0001; Pearson's correlation). Consistent with our previous findings in a different tumor model,^31^ these results demonstrate that regulation of *Bmi1* expression by *Twist1* also occurs in the leukemic blasts of AML.

### Twist1 overexpression confers a survival advantage in AML cells

**Figure 2:**
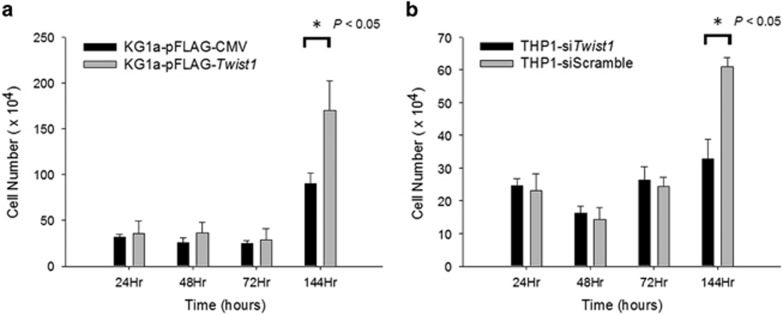
Investigation of effects of altered Twist1 expression on cellular growth of AML cells. (**a**) Effects of *Twist1* upregulation in KG1a cells. The KG1a cells were transiently transfected with either a pFLAG-*Twist1* plasmid or an empty vector, and the number of surviving cells were counted using trypan blue and recorded at 24, 48, 72 and 144 h. (**b**) Effects of *Twist1* downregulation in THP1 cells. The THP1 cells were transiently transfected with either *Twist1*-targeted siRNA or scrambled control, and the number of surviving cells were counted using trypan blue and recorded at 24, 48, 72 and 144 h. Each value is the mean±s.d. of three independent experiments.

**Figure 3:**
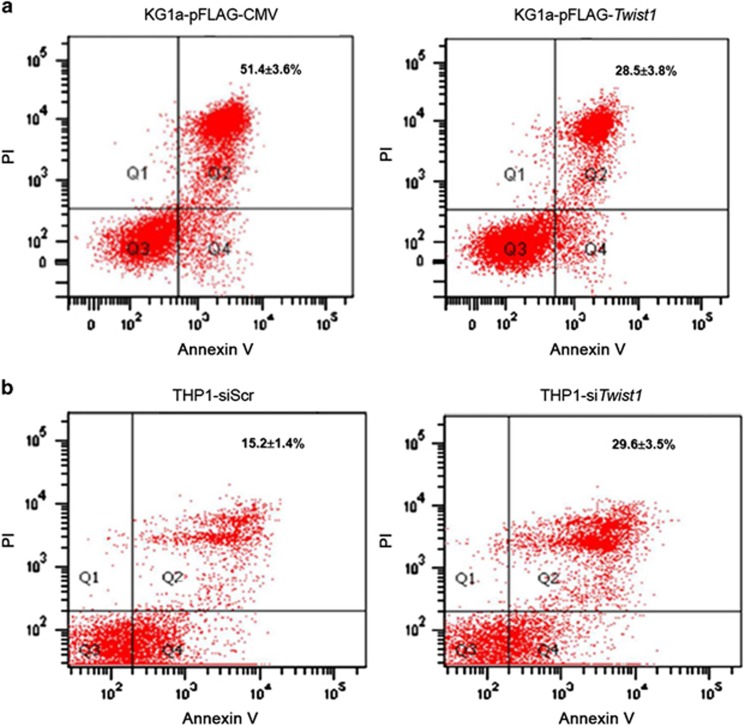
Investigation of effects of altered Twist1 expression on apoptosis. (**a**) Flow cytometric analysis using propidium iodide and Annexin V staining for apoptosis in KG1a cells 48 h after transfection with either a pFLAG-*Twist1* plasmid or an empty vector. (**b**) The proportion of apoptotic cells in THP1 cells transiently transfected with either *Twist1*-targeted siRNA or scrambled control. The percentages of apoptotic cells were expressed as the mean±s.d. of three independent experiments.

To determine whether *Twist1* overexpression alters the phenotypes of AML, we evaluated the surface antigen expression and measured the growth rates in leukemic cells with differential *Twist1* expression. Altered *Twist1* level did not lead to changes in immunophenotypes of AML cells (data not shown). On the other hand, KG1a cells transfected with *Twist1* outgrew their control counterparts after 6 days in continuous culture (Figure 2a), whereas THP1 cells treated with *Twist1* siRNA exhibited a lower growth rate than those transfected with scramble control (Figure 2b). To explore further, we examined the effects of *Twist1* on apoptosis. As shown in Figure 3a, *Twist1*-transfected KG1a cells had decreased apoptotic death compared with empty vector-transfected cells, as revealed by Annexin V and propidium iodide staining. On the contrary, downmodulation of *Twist1* in THP1 cells led to increased apoptotic ratio (Figure 3b). The data suggest that upregulated *Twist1* confers a survival advantage in the AML cells, probably by rendering them less prone to apoptotic death.

### Twist1-overexpressing AML patients exhibit unique clinical characteristics

To test the clinical significance of *Twist1* overexpression in patients with AML, we divided our AML patient cohort into two distinct groups based on the expressional status of *Twist1* and compared their baseline characteristics. Bone marrow samples from six adult control individual were assessed for their *Twist1* expression levels by real-time RT-PCR. AML patients whose leukemic blasts harbored *Twist1* expression level higher than that of the mean value of control samples by real-time RT-PCR were categorized into *Twist1*(+) group, whereas the remaining patients were subgrouped as *Twist1*(−). Among 41 AML patients, *Twist1* was overexpressed in 22 of them (53.7%). As shown in Table 1, there were no differences in patient's age, gender, hemogram at diagnosis, serum levels of lactate dehydrogenase (LDH) and uric acid, bone marrow cellularity, marrow blast percentage, degree of marrow fibrosis and prevalence of extramedullary involvement by leukemia between these two groups. Patients with overexpressed *Twist1* did have lower serum level of albumin than those without (2.8 vs 3.4 g/dl, *P*=0.024). By taking a closer look, we found that the pretreatment platelet count was lower, whereas the percentage of blasts in peripheral blood (PB) as well as the serum LDH level was higher in *Twist1*-overexpressed patients, although the differences between these two groups were not statistically significant.

We next divided PB blast percentage, PB absolute neutrophil count, platelet count and serum level of LDH into respective dichotomous variables based on clinically meaningful cutoff levels. The results are listed in Table 1 as well. Compared with those with low *Twist1* activity, AML patients with high *Twist1* expression were more likely to have PB blasts ⩾30%, and there also was a greater chance for them to be thrombocytopenic (platelet count <150 × 10^9^/l) at diagnosis. Furthermore, there were trends for those *Twist1*-overexpressing patients to have LDH levels >1.5 times upper normal limits as well as absolute neutrophil count levels below 0.5 × 10^9^/l. These clinical parameters indicate that AML patients with high *Twist1* activity might have a more aggressive disease.

### Twist1 overexpression is associated with some good prognostic features in AML patients

Table 2 showed the results of different cytogenetic and molecular risk stratification between the two groups of patients. Most of our patients had intermediate-risk cytogenetics. *Twist1* was overexpressed in both two patients with favorable cytogenetic change, whereas only one of the four adverse-risk patients exhibited upregulated *Twist1*. There was no significant difference in these two groups of patients with regard to presence of *NPM1* and *FLT3* mutations. We next incorporated cytogenetic and molecular data together and categorized our patients into different risk groups according to the European LeukemiaNet recommendations.^5^ We found that *Twist1* overexpression was less commonly seen in patients with adverse cytogenetic/molecular features. This was a clear contrast to those in good-risk groups, as all five patients in this category had upregulated *Twist1* in their leukemic blasts (Table 2, *P*=0.031).

### Twist1 overexpression is correlated with better treatment response and superior survival outcome in AML patients receiving standard treatment

**Figure 4:**
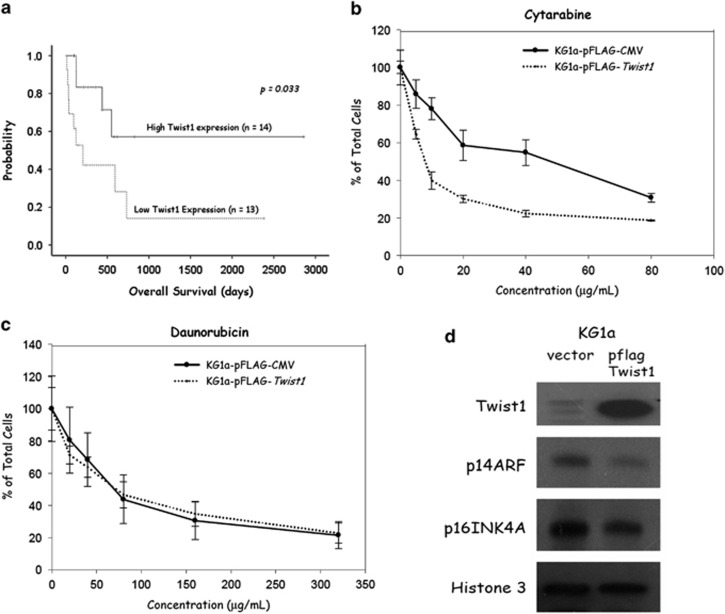
Impacts of *Twist1* overexpression on phenotypes of clinical patients and AML cell lines. (**a**) Kaplan–Meier estimates of overall survival (OS) for AML patients receiving standard treatment according to *Twist1* gene expression level. Patients with higher *Twist1* expression had significantly longer OS than those with lower expression (*P*=0.033, log-rank test). (**b** and **c**) Roles of *Twist1* upregulation on the cellular susceptibility to chemotherapeutic compounds and expression levels of downstream proteins in AML cells. After transient transfection with either a pFLAG-*Twist1* plasmid or an empty vector for 48 h, the KG1a cells were exposed to various concentrations of cytarabine (**b**) and daunorubicin (**c**) for 72 h for viability assay. The numbers of surviving cells were counted using trypan blue, and the survival curves were plotted. Each value represents the mean±s.d. of three independent experiments. (**d**) Cellular levels of Bmi1, p14ARF and p16INK4A proteins linked to altered expression of Twist1 in KG1a cells. The KG1a cells, expressing low endogenous *Twist1*, were transiently transfected with either a pFLAG-*Twist1* plasmid or an empty vector for 48 h, and the nuclear lysates were subjected to western blotting analysis. Histone 3 was used as a loading control. Representative data from three independent experiments are presented.

Among our patient cohort, 14 patients were given supportive care only because of advanced age, poor performance, presence of multiple co-morbidities or their unwillingness to receive aggressive treatment. The other 27 patients were treated with standard induction chemotherapy followed by adequate consolidation therapy per our institutional guideline. We tested the potential effects of *Twist1* overexpression on the clinical outcome of those 27 AML patients. Patients with upregulated *Twist1* were more likely to achieve CR after induction chemotherapy than those without (13/14 vs 6/13, *P*=0.013; Table 2). For those attaining CR, *Twist1*-overexpressing AML patients did so with fewer cycles of induction chemotherapy than those with low *Twist1* activity (mean cycles of chemotherapy to CR: 1.1±0.3 vs 1.8±1.2, respectively, *P*=0.037, Table 2). The observation of *Twist1*-imposed clinical benefits could be extended to survival outcome, as *Twist1*-overexpressing AML patients had a significantly longer overall survival than their counterparts did (log-rank test, *P*=0.033, Figure 4a).

### Increased susceptibility of leukemic blasts harboring overexpressed Twist1 to cytarabine cytotoxicity

To delineate the reason behind a better treatment response and a superior survival outcome conferred by *Twist1* overexpression in AML patients, we next used AML cell lines to access their vulnerability to the cytotoxicity of either cytarabine or daunorubicin, the two standard chemotherapeutic agents used in combination during induction treatment for AML patients. Compared with empty vector-transfected cells, *Twist1*-overexpressing KG1a cells were more susceptible to the treatment of cytarabine, as shown by a lower IC_50_ value (Figure 4b). Nevertheless, the cells' sensitivity to daunorubicin cytotoxicity did not exhibit apparent disparities with the differential expression levels of *Twist1* (Figure 4c). Looking further, we found that p14ARF and p16INK4A, two cell cycle regulators downstream of Twist1 transcriptional activity,^31^ were decreased in *Twist1*-overexpressing KG1a cells (Figure 4d). These data imply that by supressing p14 and p16 expression, Twist1 promotes cell cycle entry and renders leukemic cells more vulnerable to the cytotoxicity of phase-specific chemotherapeutic agent cytarabine. As a result, AML patients whose leukemic blasts harbored overexpressed *Twist1* exhibited a higher response rate to treatment and a superior survival outcome.

## Discussion

*Twist1* has been implicated in several molecular pathways that are engaged in tumor progression, apoptosis and EMT.^27, 28, 29, 30, 31^ Recent studies have also shown that its dysregulation is involved in the pathophysiology of myelodysplastic syndrome and chronic myeloid leukemia,^32, 33, 38^ two myeloid neoplasms that share similar ancestral background with AML in the hierarchy of hematopoietic differentiation. However, the role of *Twist1* activation in AML is elusive. Here we demonstrate that the *Twist1*–*Bmi1* axis is critical in the biology of AML as well. The regulation of chromatin modifier *Bmi1* by *Twist1* was confirmed in AML cells enforced with *Twist1* overexpression and indirectly affirmed by the strong positive correlation between their expression levels in primary AML samples. *In vitro* assay showed *Twist1* conferred AML cells a higher proliferation potential and increased apoptosis resistance, while clinical data suggested it caused a more aggressive disease phenotype. Strikingly, AML patients with high *Twist1* expression had a more favorable clinical course, as exhibited by a higher chance of remission induction success and a longer overall survival. This was considered to be the results of increased susceptibility of leukemic blasts to cytarabine chemotherapy, as AML cells with *Twist1* upregulation were confirmed to be more sensitive to the toxicity of cytarabine *in vitro*.

There have been some reports on the *Twist1/Bmi1*-associated aggressiveness in myeloid neoplasms. In chronic myeloid leukemia, patients whose leukemic cells harbor increased *Twist1* are more likely to fail therapies with tyrosine kinase inhibitors.^33^ Similarly in myelodysplastic syndrome, the expression levels of *Bmi1* are higher in those with advanced-stage diseases, and it predicts a potential of earlier progression in low-risk patients.^21^ Our *Twist1*-overexpressing AML patients exhibited a more aggressive clinical phenotype as well, as a more significant proportion of these patients were thrombocytopenic and/or had a PB blast count >30%. There were also trends for them to have an absolute neutrophil count below 0.5 × 10^9^/l and a high LDH level >1.5 times upper normal limit. The high PB blast counts and LDH levels suggest the aggressiveness of *Twist1*-associated AML, whereas the thrombocytopenia and neutropenia probably represent a poorer marrow reserve in *Twist1*-overexpressing AML patients. To confirm the clinical observation of Twist1-associated aggressive phenotypes, we used *in vitro* AML cell line models to demonstrate that *Twist1* endued leukemic cells more proliferation potential and more resistance to apoptosis.

Our findings of *Twist1/Bmi1*-associated apoptosis resistance echoed several previous reports in AML and myelodysplastic syndrome/AML.^18, 32, 38^ Rizo *et al.*^18^ showed that *Bmi1* expression protects AML cells against oxidative stress in a series of elegantly designed experiments. By downmodulating *Bmi1* in primary AML CD34^+^ cells, they found that progenitor and stem cell frequencies were reduced, and this was associated with increased expression of *p14ARF* and *p16INK4A* as well as an enriched level of intracellular reactive oxygen species. As a result, there was enhanced apoptosis in these cells.^18^ Convincingly, they showed that treatment with antioxidant *N*-acetyl cysteine in those *Bmi1*-downregulated AML cells led to decreased reactive oxygen species accumulation and restored progenitor frequencies, which further confirmed the *Bmi1*-conferred protective effects of leukemic blasts against apoptosis. Similarly, in the studies by Li *et al.*,^32, 38^ downmodulation of *Twist1* in KG1a cells was associated with increased apoptosis. Through immunoprecipitation, they demonstrated that endogenous *Twist1* interacted with p53 in KG1a cells. In cells treated with *Twist1* siRNA, there were increased levels of the pro-apoptotic proteins BID and BAX as well as enhanced nuclear factor-κB and p53 activities.^32, 38^ Together, these data and our results concordantly substantiate a prominent role of *Twist1*/*Bmi1* axis in the pathophysiology of AML.

Chowdhury *et al.*^20^ reported an adverse prognostic outcome of high *Bmi1* expression in patients with AML, which was contrary to our results that showed the association between *Twist1* upregulation and improved overall survival in those patients. The discrepancies on the predictive value of *Twist1*/*Bmi1* axis probably lie in the difference of treatment regimen. Repetitive courses of high-dose cytarabine (HDAC, 2–3 g/m^2^ for six doses) have been an integral part of postremission therapy in our institute. Fourteen out of the 19 AML patients (74%) who achieved CR1 in our cohort were medically fit and received HDAC as consolidation treatment. On the other hand, only 9% (6 out of 64) of the patients in the series by Chowdhury *et al.*^20^ received either HDAC or stem cell transplantation as consolidation therapy. Our *in vitro* data provide an appropriate explanation by demonstrating that *Twist1*-overexpressing AML cells are more vulnerable to the cytotoxicity of phase-specific cytarabine through suppression of cell cycle regulator p16. The finding on *Twist1*-mediated p16 suppression was parallel to previous report demonstrating that *Twist1* overexpression could lead to inhibition of INK4A/ARF activity in cancer cells.^39^ Similarly, convincing evidence has shown that the Polycomb-group protein Bmi1 controlled cell cycle propagation through repression of p14 and p16 expression.^13, 40^ In contrast, altered expressional level of *Twist1* in leukemic cells did not affect their sensitivity to daunorubucin. Our results suggest that HDAC, instead of anthracycline, could be more beneficial in AML patients with higher *Twist1* expression.

Using European LeukemiaNet-defined AML risk grouping, we find that *Twist1* overexpression is most commonly associated with good-risk patients (5/5, 100%), but it is less frequently seen in patients with poor molecular/cytogenetic risk (2/8, 25%). The mechanism underlying such an association is not immediately clear. In spite of the facts that the expression of *Bmi1* could be regulated by *Twist1* and that *Bmi1* is constantly associated with the ‘stemness' properties of AML and hematopoietic cells, *Twist1*, as a transcriptional factor, might affect leukemogenesis through other unidentified pathways that are independent of *Bmi1* activity. It is plausible that *Twist1* interacts with the molecular signaling pathways used by those good-risk AML subtypes and coordinately confers them a survival advantage. Therefore, a multivariate analysis needs to be carried out to confirm the unequivocal prognostic value of *Twist1* in AML. Unfortunately, we are unable to do so because of the limited case number in our cohort.

In conclusion, the current study provides strong evidence for a novel and crucial role of *Twist1* in the pathophysiology of a subset of AML patients that leads to unique clinical phenotypes. The data also add values to the complex picture of molecular pathogenesis in AML. Although further elucidation and molecular dissection is mandatory, our results indicate that *Twist1* could represent a powerful biomarker that serves as a prognostic as well as therapeutic guide for patients with AML.

## Figures and Tables

**Table 1:** Clinical and laboratory features of 41 patients with AML, stratified by *Twist1* expression level

	*Twist1(+) (*n=*22)*	*Twist1(−) (*n=*19)*	P-*value*
Age (years)	58.1±16.9	62.5±17.6	0.410
Male, *n* (%)	13 (59.1%)	11 (57.9%)	1.000
WBC (× 10^9^/l)	55.7±75.5	38.4±74.1	0.466
Hemoglobin (g/dl)	7.9±2.3	8.7±2.5	0.325
Platelet count (× 10^9^/l)	47±36	129±206	0.103
Platelet count <150 × 10^9^/l, *n* (%)	22 (100%)	14 (73.7%)	**0.016**
ANC<0.5 × 10^9^/l, *n* (%)	6 (27.3%)	1 (5.3%)	0.099
PB myeloblast ⩾30%, *n* (%)	17 (77.3%)	7 (36.8%)	**0.012**
LDH (U/dl)	1071±1976	461±666	0.223
LDH<1.5 × UNL, *n* (%)	4 (28.6%)	10 (55.6%)	0.086
Uric acid (g/dl)	6.9±4.4	7.0±2.9	0.938
Albumin (g/dl)	2.8±0.7	3.4±0.6	**0.024**
BM cellularity (%)	86.0±15.4	88.9±14.5	0.541
BM blast (%)	76.0±23.7	72.9±20.6	0.665
BM fibrosis, nil or mild, *n* (%)	19 (90.5%)	13 (81.3%)	0.634
Extramedullary involvement (+), *n* (%)	4 (18.2%)	3 (15.8%)	1.000

Abbreviations: AML, acute myeloid leukemia; ANC, absolute neutrophil count; BM, bone marrow; LDH, lactate dehydrogenase; PB, peripheral blood; UNL, upper normal limit; WBC, white blood cell count. Values are reported as mean±s.d. unless otherwise indicated. *P*-values with statistically significant differences are shown in bold.

**Table 2:** Cytogenetic/molecular risk and treatment outcome of 41 patients with AML, stratified by *Twist1* expression level

	*Twist1 (+)*	*Twist1 (−)*	P*-value*
*Cytogenetic risk*			0.222
Good	2	0	
Intermediate	15	16	
Poor	1	3	
			
*NPM1* *mutation*			0.464
Mutated	7	3	
Wild type	15	15	
			
*FLT3* *mutation*			1.000
Mutated	5	4	
Wild type	17	14	
			
*Molecular/cytogenetic risk*			**0.031**
Good	5	0	
Intermediate	15	13	
Poor	2	6	
			
*Achievement of CR*			**0.013**
Yes	13	6	
No	1	7	
			
No. of chemo cycles to CR	1.1±0.3	1.8±1.2	**0.037**

Abbreviations: AML, acute myeloid leukemia; CR, complete remission. *P*-values with statistically significant differences are shown in bold.
